# Splitting of the magnetic encephalogram into «brain» and «non-brain» physiological signals based on the joint analysis of frequency-pattern functional tomograms and magnetic resonance images

**DOI:** 10.3389/fncir.2022.834434

**Published:** 2022-08-26

**Authors:** Rodolfo R. Llinás, Stanislav Rykunov, Kerry D. Walton, Anna Boyko, Mikhail Ustinin

**Affiliations:** ^1^Department of Neuroscience, Center for Neuromagnetism, New York University Grossman School of Medicine, New York, NY, United States; ^2^Keldysh Institute of Applied Mathematics, Russian Academy of Sciences, Moscow, Russia

**Keywords:** magnetic encephalography, frequency-pattern analysis, functional tomography, extraction of partial spectra, time series reconstruction

## Abstract

The article considers the problem of dividing the encephalography data into two time series, that generated by the brain and that generated by other electrical sources located in the human head. The magnetic encephalograms and magnetic resonance images of the head were recorded in the Center for Neuromagnetism at NYU Grossman School of Medicine. Data obtained at McGill University and Montreal University were also used. Recordings were made in a magnetically shielded room and the gradiometers were designed to suppress external noise, making it possible to eliminate them from the data analysis. Magnetic encephalograms were analyzed by the method of functional tomography, based on the Fourier transform and on the solution of inverse problem for all frequencies. In this method, one spatial position is assigned to each frequency component. Magnetic resonance images of the head were evaluated to annotate the space to be included in the analysis. The included space was divided into two parts: «brain» and «non-brain». The frequency components were classified by the feature of their inclusion in one or the other part. The set of frequencies, designated as «brain», represented the partial spectrum of the brain signal, while the set of frequencies designated as «non-brain», represented the partial spectrum of the physiological noise produced by the head. Both partial spectra shared the same frequency band. From the partial spectra, a time series of the «brain» area signal and «non-brain» area head noise were reconstructed. Summary spectral power of the signal was found to be ten times greater than the noise. The proposed method makes it possible to analyze in detail both the signal and the noise components of the encephalogram and to filter the magnetic encephalogram.

## Introduction

Source analysis is a key element in the interpretation of magnetoencephalographic (MEG) recordings. Such analysis can reveal, not just the origin of a signal within the brain, but temporal and spatial patterns of brain activity. Such patterns can be related to behavior providing a functionally meaningful recording (see Gross, 2019). However, one of the main problems in the reconstruction of the sources of brain activity from MEG data is that the experimental data contain many noise sources. The noise sources that can be distinguished include those: (a) intrinsic to the encephalographic instrument itself; (b) from the environment, subways for example, and (c) with a biological origin such as the heartbeat.

The intrinsic noises of a magnetic encephalograph are due to the internal structure of the encephalograph and to the processes taking place within the instrument (Ryhanen et al., 1989; Hämäläinen et al., 1993). Usually, the level of these noises is small compared to the signal under investigation, and their nature is random. However, when examining high-frequency brain signals, clearing the data of the intrinsic noise of the encephalograph can significantly improve the signal-to-noise ratio. Several methods have been introduced to automatically reduce such noise (Jas et al., 2017; Mutanen et al., 2018). A denoising method based on the construction of spatial and temporal correlations between the measurement channels and the extraction of random noise was proposed in de Cheveigné and Simon (2008a) and Larson and Taulu (2018). Clarke proposed a joint use of these methods (Clarke et al., 2020).

The main sources of environmental noise are various electrical devices located both near the encephalographic instrument and those far from it. Urban transport is an important source of interference, generating electromagnetic interference and vibrations (Hansen et al., 2010). To reduce the noise from the environment the following techniques have been used: (a) magnetically shielded rooms (Kelha, 1981; Mager, 1981; Bork et al., 2000; Cohen et al., 2002); (b) systems for active compensation of external magnetic fields (Okada et al., 2016; Sun et al., 2018; Iivanainen et al., 2019); and (c) software and hardware solutions incorporated in the design of encephalographs (Vrba et al., 1995; Taulu et al., 2004; Taulu and Simola, 2006; Vrba and Wilson, 2007). These methods, applied individually or in combination, can reduce the level of external interference by 60–30000 times (Sun et al., 2018).

The strongest sources of artifacts of biological origin are the heartbeat (Jousmäki and Hari, 1996), breathing (Rodin et al., 2005), and face and eye muscle activity (Hansen et al., 2010). To clean the data of these artifacts, researchers have used methods based on the analysis of independent components (ICA) (Sarela and Valpola, 2005; Escudero et al., 2007, 2011; Breuer et al., 2014a,b), spectral signal space projection (Ramírez et al., 2011) and spatial signal separation (Taulu et al., 2004; de Cheveigné and Simon, 2008b). These methods work well when the spatial patterns of artifacts are stationary or only change slightly during the experiment. To effectively suppress non-stationary artifacts, methods have been proposed based on identifying patterns of artifacts in each time window using additional data obtained from an electrocardiogram (Adachi et al., 2001; Tal and Abeles, 2013; Sun et al., 2016). A method based on the use of convolutional neural networks was proposed to isolate and filter eye-blink artifacts (Garg et al., 2017).

Noise is not the only problem in the reconstruction of activity sources. For example, a closely located strong source may interfere with the localization of a weaker source. The construction of an adaptive beamformer for isolating a weak response signal using the recordings of the control state has been described (Sekihara et al., 2006). The use of *a priori* knowledge about stimulus and response to study the activity of the subcortical structures of the brain has also been considered (Krishnaswamy et al., 2017).

This study is the further development of the method of frequency-pattern analysis to decompose complex systems into functionally invariant entities (Llinás and Ustinin, 2014, 2016). This method makes it possible to address general spectra to the partial spectra of static functional entities and to restore their time series. The method is based on the complete utilization of the long-time series, while the multichannel nature of the data is also completely accounted for making it possible to extract elementary sources of the brain activity. It was successively applied in alpha-rhythm studies (Llinás et al., 2015a) and in partial spectroscopy of the brain (Llinás et al., 2015b; Rykunov et al., 2016). Many elementary sources located outside the brain contribute physiological noises to magnetoencephalographic (MEG) data. It was found that the processing of MEG data by the frequency-pattern analysis allows an estimate of the functional structure of the head as a whole, including signals generated by the brain, face, neck and other sources (Llinás et al., 2020). The aim of the present study was to divide the MEG signal into those generated by the brain and those generated by non-brain areas.

## Methods

### Magnetoencephalographic and magnetic resonance imaging recoding methods

The data was obtained from 10 healthy adult subjects. Recordings from seven subjects were carried out at the NYU Grossman School of Medicine Center for Neuromagnetism located at the Bellevue Hospital Center. These comprised 5 men and 2 women, 27 to 41.5 years of age (mean 33.1 ± 1.59 years, median 33.4 years.). An informed written consent was obtained from each subject before the recordings were made in accordance with the Declaration of Helsinki. The NYU Institutional Review Board approved the study.

All MEG recordings were obtained while the subject was seated inside a mu-metal magnetic shielded room using a 275-channel whole-head MEG instrument (CTF Systems Inc., Port Coquitlam, BC, Canada). Artifacts and distant noise were reduced using a 3rd order gradientometer (McCubbin et al., 2004).

The location of the subject’s head, within the recording helmet, was monitored at the beginning and end of each run using electrodes attached to the three fiducial marker points (nasion, left and right pre-auricular points). The same fiducial marker points were used during the magnetic resonance imaging (MRI) allowing for co-registration of the MEG and MRI data.

During each 5-min MEG recording run, magnetic fields were obtained in 30 consecutive 10-s trials. This procedure allowed the removal of recorded segments, in cases where the subject moved during any of the recording trials, as well as other possible recording artifacts. Three recording runs were obtained from each subject, two with the eyes closed, which minimized signals from ocular muscles and visual system activation. During one run the eyes were kept open. Under this condition the alpha range frequency (8–13 Hz) is normally decreased. One recording for each subject under the eyes-closed condition was used in this data analysis.

Magnetic resonance imaging scans at NYU were carried out on 1.5 T Allegra Siemens platform. A degree of uniformity was maintained across subjects by performing MRI constrained MFT modified minimum norm inverse modeling on each data set.

Three datasets (MEG+MRI from 2 men (21 and 30 years of age) and one woman (23 years of age) were obtained from an open MEG archive OMEGA (Niso et al., 2015). These data were recorded at McGill University and Montreal University using an MEG instrument similar to that at NYU under similar conditions. Please see Niso et al. (2015) for MEG and MRI recording information on the three subjects from the OMEGA archive.

### Frequency-pattern functional tomography

Magnetic fields were recorded using an MEG system, consisting of *K* sensors (channels), which provided the set of experimental vectors {**b_k_**}, *k* = 1,…,*K*. This approach discretely samples set of continuous functions $\left\{{\tilde{B}}_{k}\,\left(t\right)\right\}-$magnetic inductions in a *K* channel set.

Consider the Fourier transform


$$a_{0\,k}=\frac{2}{T}\,\int_{0}^{T}{\tilde{B}}_{k}\,\left(t\right)\,dt,a_{n\,k}=\frac{2}{T}\,\int_{0}^{T}{\tilde{B}}_{k}\,\left(t\right)\,c\,o\,s\,\left(2\,\pi \,\nu_{n}\,t\right)\,dt,b_{n\,k}$$

(1) $$=\frac{2}{T}\,\int_{0}^{T}{\tilde{B}}_{k}\,\left(t\right)\,s\,i\,n\,\left(2\,\pi \,\nu_{n}\,t\right)\,dt,$$

where *a*_0*k*_,*a*_*nk*_,*b*_*nk*_ are Fourier coefficients for the frequency ν_*n*_ in the channel array *k*, and *n* = 1,…,*N*,*N* = ν_*max*_*T*,where ν_*max*_ is the highest desirable frequency. The coefficient *a*_*0k*_ is not considered hereafter, given that the constant field component has no meaning in superconducting quantum interference device (SQUID) sensor measurement sets. Given high sampling frequency (1,200 Hz in our experiments), vectors {**b**_*k*_} represent continuous functions $\left\{{\tilde{B}}_{k}\,\left(t\right)\right\}$ with sufficient precision, such that integrals (1) can be effectively calculated using discrete Fourier transform (Frigo and Johnson, 2005).

The frequency resolution is determined by *T*: $△\,\nu =\nu_{n}-\nu_{n-1}=\frac{1}{T}$. This implies that to reveal the detailed frequency structure of the system, it is necessary that: (1) Data be recorded for a sufficient time; and (2) That calculations are made for all spectra for the whole duration of the recording procedure time *T*. In our experiments *T* was equal to 300 s, thus providing a frequency resolution of 0.0033 Hz.

The next step of the analysis requires the restoring of the multichannel signal at every frequency and the analysis of the functions obtained. The multichannel signal is restored at frequency ν_*n*_ in all channels:


(2) $$B_{n\,k}\,\left(t\right)=\rho_{n\,k}\,\sin\left(2\,\pi \,\nu_{n}\,t+\varphi_{n\,k}\right),$$

where $\rho_{n\,k}=\sqrt{a_{n\,k}^{2}+b_{n\,k}^{2}},$φ_*nk*_ = *atan2*(*a*_*nk*_,*b*_*nk*_), and *a*_*nk*_,*b*_*nk*_ are Fourier coefficients, found in equation (1).

The proximity of phases φ_*nk*_ in different channels can be characterized by the value of coherence (Llinás and Ustinin, 2014):


(3) $$C_{1\,f}=1-\frac{\min_{t\in |0,T_{\nu_{n}}|}\sum_{k=1}^{K}B_{n\,k}^{2}\,\left(t\right)}{\max_{t\in |0,T_{\nu_{n}}|}\sum_{k=1}^{K}B_{n\,k}^{2}\,\left(t\right)},$$

where min and max are calculated at the period *T*_*_ν n_*_ of frequency ν_*n*_. If all channels have equal phases φ_*nk*_ = φ_*n*_ at frequency ν_*n*_, then *C*_*1f*_ is equal to 1. If φ_*nk*_ = φ_*n*_, then equation (2) describes a coherent multichannel oscillation and can be written as


(4) $$B_{n\,k}\,\left(t\right)=\rho_{n\,k}\,\sin\left(2\,\pi \,\nu_{n}\,t+\varphi_{n}\right)={\hat{\rho }}_{n\,k}\,\rho_{n}\,\sin\left(2\,\pi \,\nu_{n}\,t+\varphi_{n}\right),$$

where $\rho_{n}=\sqrt{\sum_{k=1}^{K}\rho_{n\,k}^{2}}$ is the amplitude, and ${\hat{\rho }}_{n\,k}=\frac{\rho_{n\,k}}{\rho_{n}}$ is the normalized pattern of oscillation.

Thus, equation (4) provides the separation of time and space. The normalized pattern makes it possible to determine the spatial structure of the source from the inverse problem solution, under the assumption that the structure is positionally stable throughout the entire period of the oscillation. The time course of the field is determined by the function ρ_*n*_*sin*⁡(2πν_*n*_*t*+φ_*n*_), which is common for all channels, i.e., this source is oscillating as a whole at the frequency ν_*n*_.

The theoretical foundations for the reconstruction of static functional entities (neuronal circuits, or sources) have been developed (Llinás and Ustinin, 2014, 2016). This reconstruction is based on a detailed frequency analysis and extraction of the frequencies that have high coherence and similar patterns.

The algorithm of mass precise frequency-pattern analysis was formulated as follows:

1. Discrete Fourier Transform of the multichannel signal for the whole recording time *T*.
2. Inverse Fourier Transform–restoration of the signal at each frequency.
3. If the coherence at the particular frequency is close to 1 [see equation (3)], then the pattern and frequency will be used as an elementary coherent oscillation. See equation (4).
4. If the restored signal consists of several phase-shifted coherent oscillations, then extract those oscillations:
  a. Apply Independent Component Analysis algorithm (see Belouchrani et al., 1997) to restored time-series;
  b. Select nonzero components;
  c. Apply direct discrete Fourier Transform to each of the selected components and calculate amplitude, normalized pattern and phase using equations 1 and 4.

After the fourth step of this analysis, the initial multichannel signal is represented as a sum of elementary coherent oscillations:


$$B_{k}\,\left(t\right)≅\sum_{n=1}^{N}\sum_{m=1}^{M}D_{m\,n}\,{\hat{\rho }}_{m\,n\,k}\,\sin\left(2\,\pi \,\nu_{n}\,t+\varphi_{m\,n}\right),\nu_{n}=\frac{n}{T},N$$

(5) $$=\nu_{m\,a\,x}\,T,m=1,\ldots ,M$$

where *M* is maximal number of coherent oscillations, extracted at the frequency ν_*n*_.

Each elementary oscillation is characterized by frequency ν_*n*_, phase φ_*mn*_, amplitude *D*_*mn*_, normalized pattern ${\hat{\rho }}_{m\,n\,k}$ and is produced by the functional entity having a constant spatial structure.

We, thus, define the “Functional Tomogram” as the electrical functional structure of the system, reconstructed from the analysis of the set of normalized patterns $\left\{{\hat{\rho }}_{m\,n}\right\}$. The functional tomogram displays a 3-dimensional map of the energy emitted by all the sources located at a given point in space. In order to build a functional tomogram, the space under study is divided into *N*_*x*_×*N*_*y*_×*N*_*z*_ elementary cubicles with centers in **r**_*ijs*_. The edge of the cubicle is selected in accordance with the desirable precision; in this study, we selected 2.0 mm. To calculate the energy produced by all the sources located at the center of a given cubicle, the set of *L* trial dipoles **Q**_*ijs*_ is built. The magnetic induction at point **r**, for dipole **Q**_*ijsl*_, located at **r**_*ijs*_, is calculated as a current dipole in a spherical conductor (Sarvas, 1987).


$$\text{B}\,\left(r\right)=-\mu_{0}\,\nabla U\,\left(r\right),U\,\left(r\right)=-\frac{1}{4\,\pi }\,\frac{\left({\text{Q}}_{i\,j\,s\,l}\times {\text{r}}_{i\,j\,s},r\right)}{F},$$

(6) $$F=a\,\left(a\,r+r^{2}-\left({\text{r}}_{i\,j\,s},r\right)\right),\text{a}=\text{r}-{\text{r}}_{i\,j\,s},\mu_{0}=4\,\pi \cdot 10^{-7}$$

All trial dipoles lie in the same plane, orthogonal to **r**_*ijs*_, as the vector product **Q**_*ijsl*_×**r**_*ijs*_ is non-zero only for those dipoles. Trial dipoles cover the circle in *L*_*max*_ directions with 360/*L*_*max*_ degrees step, in this study *L*_*max*_ = 24. The set of normalized trial patterns is then calculated:


(7) $$\left\{{\hat{\rho }}_{i\,j\,s\,l}^{t\,r}\right\},i=1,\ldots ,N_{x};j=1,\ldots ,N_{y};s=1,\ldots ,N_{z};l=1,\ldots ,L_{m\,a\,x}$$

In this study more than 25 million trial patterns were used for each object, calculated at the grid, covering the space of the experiment.

For each normalized pattern ${\hat{\rho }}_{m\,n}$ from equation (5), the following function was calculated, giving the difference between this pattern and one of the actual trial patterns:


(8) $$\chi \,\left(i,j,s,l\right)=\sum_{k=1}^{K}{\left({\hat{\rho }}_{i\,j\,s\,l\,k}^{t\,r}-{\hat{\rho }}_{m\,n\,k}\right)}^{2}$$

The position and direction of the source producing the experimental pattern ${\hat{\rho }}_{m\,n}$ were determined by numbers (*I*,*J*,*S*,*L*), providing the minimum to the function χ(*i*,*j*,*s*,*l*) over the variables, *i* = 1,…,*N*_*x*_;*j* = 1,…,*N*_*y*_; = *s* = 1,…,*N*_*z*_;*l* = 1,…,*L*_*max*_. The energy of this source $D_{m\,n}^{2}$ is added to the energy produced from the cubicle with the center at **r**_*ijs*_. Following this procedure for all normalized patterns ${\hat{\rho }}_{m\,n}$: *m* = 1,…,*M*;;*n* = 1,…,*N*, it is possible to determine the energy space distribution of all oscillations from equation (5).

The result of such distribution is then the Functional Tomogram of the system under study.

### Simulation/forward modeling

To check localization accuracy the following computational experiment was conducted:

1. MRI image and co-registered gradientometer were selected from data recorded at NYU.
2. From MRI image voxels composing the head were selected and formed a “head mask.”
3. A total of 5000 test dipole sources were randomly distributed in the “head mask” space. For each of the dipoles its unique frequency and random dipolar moment were set. Dipole amplitudes varied in range 10–100 nAm, frequencies were in 1–17.667 Hz band with 0.033 Hz step.
4. Simulated MEG recordings were reconstructed for each test source, using single shell model (see equation 5), then summary simulated MEG was calculated. Sampling frequency was set to 1,200 Hz, single trial length was set to 300 s.
5. Functional tomogram was calculated as described in section “Frequency-pattern functional tomography.”

Then results of inverse problem solution were compared to known locations of sources, set on step 3. No shift in locations and directions of sources was found. Similar computation experiment with “virtual magnetometer” was conducted. To create “virtual magnetometer” we’ve used channel positions and orientations of the inner layer of CTF MEG 275 sensors. No shift in locations and directions of sources was found. It can be concluded that the proposed method works precisely in the analysis of clean simulated data.

### Partial spectroscopy of the head and brain

A partial spectrum is understood as a set of frequencies and Fourier transform coefficients belonging to sources located in a given region of space. The basic principles and method for calculating partial spectra have been outlined (Llinás et al., 2015b; Rykunov et al., 2016). The first step in calculating such spectra is the segmentation of a magnetic resonance image (MRI)–the anatomical structure of the brain of a given subject. For this purpose, we used the Freesurfer software (Fischl, 2004; Fischl et al., 2004; Desikan et al., 2006),^1^ which allows segmentation in automatic mode. The result of MRI segmentation is an annotated three-dimensional map of the brain in which each voxel of the magnetic resonance image is associated with its belonging to one or another part of the brain. Then, binary voxel masks of the selected sections are constructed from the annotated maps–all voxels (volume elements) related to the selected section have a value of 1, the rest have a value of 0. The downsampling procedure is applied to the resulting masks to match the spatial resolution of the masks to the spatial resolution of the functional tomogram. If, after downsampling, the constructed masks for different segments contain common voxels, these voxels are removed from all masks.

At the third step, voxel masks are converted into index form–each non-zero voxel is assigned its ordinal number in the three-dimensional array. At the fourth step those experimental patterns are selected whose index coordinates are equal to the index coordinates of the voxels of the mask of the section under consideration. The frequencies and Fourier coefficients of these patterns form a partial spectrum of the considered section of the head or brain. If the frequencies are classified between two areas «brain» and «non-brain», they can be defined by the two following functions:


(9) $$f^{\left(b\right)}\,\left(n\right)=\left\{ \begin{array}{c} 1,i\,f\,\nu_{n}\,b\,e\,l\,o\,n\,g\,s\,t\,o\,b\,r\,a\,i\,n\\ 0,i\,f\,\nu_{n}\,b\,e\,l\,o\,n\,g\,s\,t\,o\,n\,o\,n-b\,r\,a\,i\,n \end{array}\right.$$

and


(10) $$f^{\left(n\,b\right)}\,\left(n\right)=\left\{ \begin{array}{c} 0,i\,f\,\nu_{n}\,b\,e\,l\,o\,n\,g\,s\,t\,o\,b\,r\,a\,i\,n\\ 1,i\,f\,\nu_{n}\,b\,e\,l\,o\,n\,g\,s\,t\,o\,n\,o\,n-b\,r\,a\,i\,n \end{array}\right.$$

The brain signal is obtained by the following Inverse Fourier transform:


(11) $$B_{k}^{\left(b\right)}\,\left(t\right)=\sum_{n=1}^{N}f^{\left(b\right)}\,\left(n\right)\,B_{n\,k}\,\left(t\right)$$

where *B*_*nk*_(*t*) is calculated using equation 2.

The non-brain signal is obtained by the following Inverse Fourier transform:


(12) $$B_{k}^{\left(n\,b\right)}\,\left(t\right)=\sum_{n=1}^{N}f^{\left(n\,b\right)}\,\left(n\right)\,B_{n\,k}\,\left(t\right)$$

Instantaneous power for brain and non-brain signals in all channels are given by:


(13) $$p^{\left(b\right)}\left(t\right)=\;\sum_{n=1}^{N}f^{\left(b\right)}\left(n\right)\cdot \sum_{k=1}^{K}B_{n\,k}^{2}\left(t\right)$$

(14) $$p^{\left(n\,b\right)}\left(t\right)=\;\sum_{n=1}^{N}f^{\left(n\,b\right)}\left(n\right)\cdot \sum_{k=1}^{K}B_{n\,k}^{2}\left(t\right)$$

The summary power for brain in channel *kisgivenby*:


(15) $$P^{\left(b\right)}\left(k\right)=\;\int_{0}^{T}\sum_{n=1}^{N}f^{\left(b\right)}\left(n\right)B_{n\,k}^{2}\left(t\right)dt$$

The summary power for non-brain in channel *k* is given by:


(16) $$P^{\left(n\,b\right)}\left(k\right)=\;\int_{0}^{T}\sum_{n=1}^{N}f^{\left(n\,b\right)}\left(n\right)B_{n\,k}^{2}\left(t\right)dt$$

Ratio of the “brain” MEG power to “non-brain” MEG power is characterized by:


(17) $$B\,N\,b\,R=\frac{\sum_{k=1}^{K}P^{\left(b\right)}\,\left(k\right)}{\sum_{k=1}^{K}P^{\left(n\,b\right)}\,\left(k\right)}$$

## Experimental results

Analysis of the data proceeded in several steps from the broadest consideration of signal vs. noise to the most detailed analysis of brain signal vs. other head signals.

**FIGURE 1:**
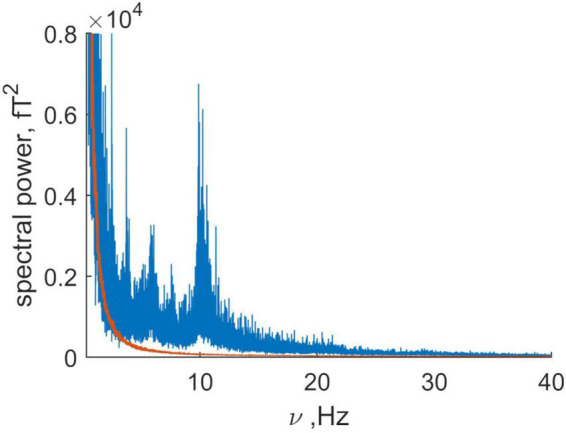
Comparison of the summary power of the magnetic recording for the spontaneous activity (blue) and the summary power for the “empty room” recording or baseline noise (orange). Note that the average signal to noise ratio is 26, being about 100 for alpha rhythm and 50 for beta rhythm. Here the signal is produced by the “brain” and “non-brain” physiological sources, while baseline noise has a non-human (mainly technical) origin.

The first step in our analysis was to distinguish the signal generated by the head from that due to the residual distant noise and the activity of the instrument itself. The general spectral features of the experimental data were characterized by using the sum of powers in all the channels:

$P\,o\,w\,e\,r\,\left(\nu_{n}\right)=\sum_{k=1}^{K}\rho_{n\,k}^{2}$, where *k* is the channel number, the number of frequency is *n*, and ρ_*nk*_ is the Fourier amplitude (2). Figure 1 is a plot of the spectral power as a function of frequency. The contribution from the subject’s head (the signal) is shown in blue and the combined signal from the instrument and far sources (the noise) is shown in orange. Brain activity in healthy adults is largest in the alpha (8–13 Hz) and beta (13–30 Hz) frequency ranges. Figure 1 shows that the alpha range signal is about 100 times that for the noise and that the beta range activity is about 50 times that of the noise. Because the brain signal is so much larger than the residual far distance noise, the latter will be neglected in further analysis (Note that the average signal to noise ratio is 26).

**FIGURE 2:**
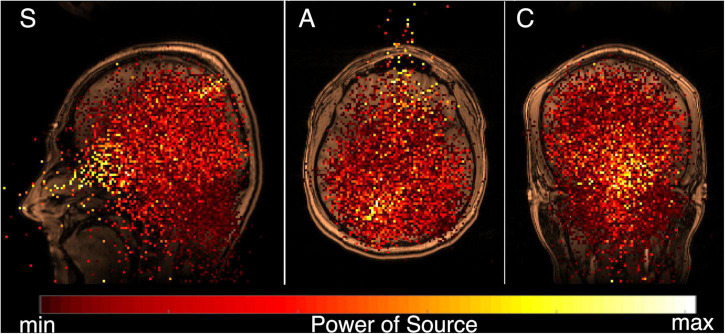
Functional tomogram for wide frequency band 0.3–100 Hz, for MEG recording made with the eyes closed, co-registered and superimposed over the subject’s MRI. Standard tomographic sections are shown; sagittal (S), axial (A), and coronal (C). A color map for the power of elementary sources is shown at the bottom. The strongest (yellow) elementary sources can be divided into two main groups–one generally corresponding the area for the alpha rhythm (see Llinás et al., 2015a) inside the brain, while the other is situated inside the head, but outside the brain.

In the next step, the spectral power of the MEG signal (0.3–100 Hz) and MRI image of each subject were co-registered and superimposed. The resulting total functional tomogram is shown in Figure 2 for one subject in the sagittal (S), axial (A) and coronal (C) planes. The inverse problem (that locates the MEG signal) for each elementary source was solved in the whole experimental space 25 cm × 25 cm × 25 cm without additional conditions. That almost all elementary sources were localized inside the head, generally confirmed the correctness of our model. Also, it indicates that the MEG data was rather clean from external noise.

The strongest (yellow) elementary sources can be divided into two main groups–one generally corresponding the area for the alpha rhythm (see Llinás et al., 2015a) inside the brain, while the other is situated inside the head, but outside the brain. Both kinds of sources were found from the analysis of one MEG, which registered all physiological activity inside and near the helmet of the instrument. The basic aim of our study was to divide the experimental MEG data into two synthetic MEGs, one produced by the «brain» sources, and another produced by the «non-brain» sources.

**FIGURE 3:**
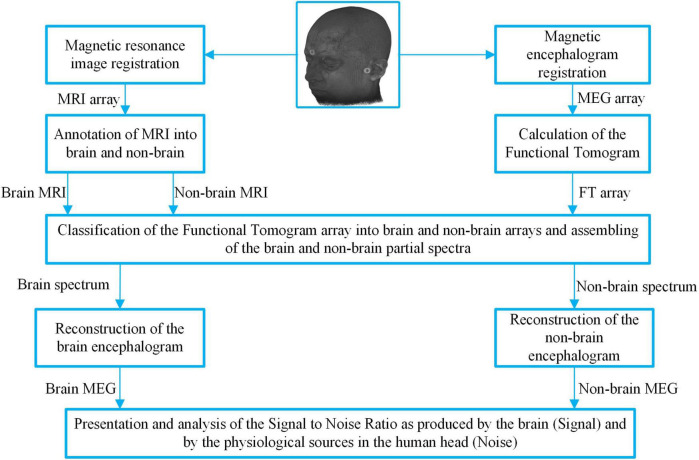
Block diagram of the partial spectroscopy algorithm and the data flow to divide the MEG signal into «brain» and «non-brain» MEGs. Markers at the fiducial points can be seen, which are providing the co-registration of the MRI and MEG and make their cooperative analysis possible. This diagram illustrates classification of the frequencies based on their localization in the annotated MRI, making it possible to compose two synthetic MEGs.

The aim of the third step was to distinguish «brain» from «non-brain» sources. To achieve this, the frequency-pattern tomography was combined with partial spectroscopy as described in the section “Methods.” The flow used in the data processing algorithm is shown in the block diagram in Figure 3 where localization information is on the left side and magnetic activity information is on the right side. The process has six steps.

**FIGURE 4:**
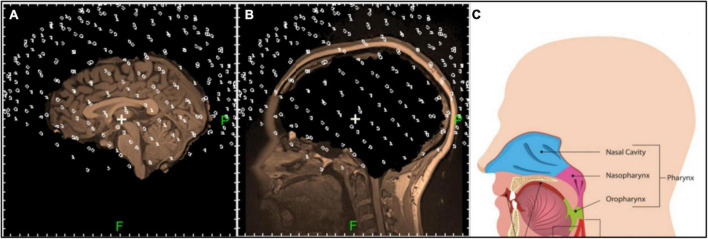
Sagittal tomographic sections of the «brain» **(A)** and «non-brain» **(B)** areas of the head after the annotation of initial MRI. Positions of the sensors are denoted with white rings. Anatomy of the possible strong non-brain sources is shown in panel **(C)**.

1. Each voxel in the MRI was assigned the feature «brain» or «non-brain» based on the anatomy (see Figures 4A,B).

2. The functional tomogram was calculated based on the MEG. This is a set of elementary oscillations with known frequencies and source origins.
3. Elementary oscillations are classified based on the feature of the origin voxel as being «brain» or «non-brain» in step 1 (Figure 3 top wide Classification box).
4. Two partial spectra were assembled. One from the frequencies of the «brain» oscillations, and the other from the frequencies of the «non-brain» oscillations.
5. The «brain» and «non-brain» signals were reconstructed from the corresponding partial spectra.
6. The properties of restored MEGs were analyzed and compared. Note that because all partial frequencies were selected from the initial spectrum the sum of those reconstructed signals was equal to the experimental MEG (Figure 3 last row).

The results of this process of dividing the MRI into «brain» and «non-brain» areas are shown in Figures 4A,B, respectively (Voxels classified into one or another category were superimposed onto the corresponding MRI image and presented as separate objects). The positions of the MEG sensors for this subject, based on the fiducial markers, are shown as small dots superimposed on the MRI images in Figures 4A,B. Non-brain structures in the head that may be strong signal sources are shown in Figure 4C. As can be seen in Figure 4, the brain was properly surrounded by the MEG sensors. The scalp was, naturally, closer to sensors than were the internal structures. The sources in the brainstem and cerebellum were registered effectively, as were strong sources in the head below the brain.

**FIGURE 5:**
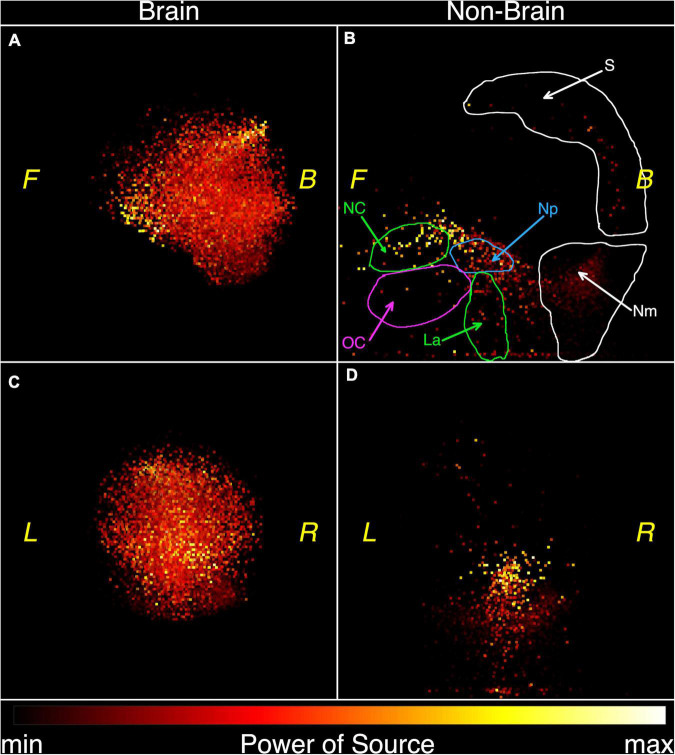
Functional tomograms of the «brain» and «non-brain» areas of the head after the classification of elementary sources, 3D-views: brain, left side view **(A)**; non-brain, left side view **(B)**; brain, frontal view **(C)**; non-brain, frontal view **(D)**. Color map for the power of elementary sources is shown at the bottom. Directions are denoted by: L, left; R, right; F, front; B, back. Anatomic details are denoted by: NC, nasal cavity; OC, oral cavity and tongue; La, larynx; Np, nasopharynx; S, scalp; Nm, neck muscles.

The functional tomograms were next viewed in three dimensions using the specialized software FTViewer (Rykunov et al., 2020). Left side and frontal views are shown in Figure 5 after the classification of elementary oscillations by the «brain» or «non-brain» feature of their source origin in one subject. The left side surface of the 3-dimensional functional tomograms of the brain is shown in Figure 5A. Several components may be distinguished in the non-brain image in Figure 5B including the scalp surface (S), nasal cavity (NC), oral cavity (OC), nasopharynx (Np), larynx (La), and the back of the neck (Nm). The frontal view of the brain is shown in Figure 5C. The corresponding non-brain areas are shown in Figure 5D where the strongest sources correspond to nasal cavity and nasopharynx. Powerful sources in the lower aspect of the image may correspond to neural/muscle activity in the nasal cavity, oral cavity, nasopharynx, oropharynx, and oral cavity seen in Figure 5B.

**FIGURE 6:**
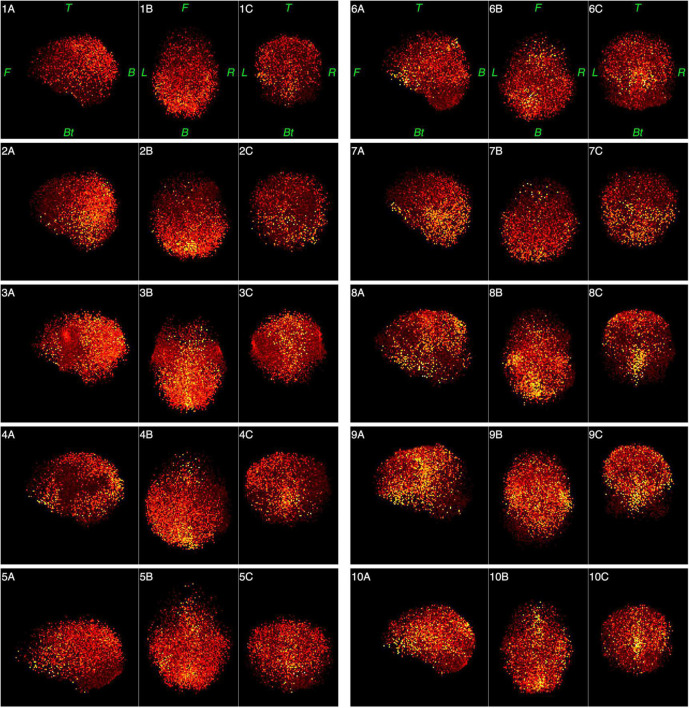
Functional tomograms for all subjects of the «brain» areas of the head after the classification of elementary sources, 3D-views: left side view (A); top view (B); frontal view (C). Color map for the power of elementary sources is shown at the bottom of Figure 5. Directions are denoted by: L, left; R, right; F, front; B, back, Bt, bottom; T, top.

**FIGURE 7:**
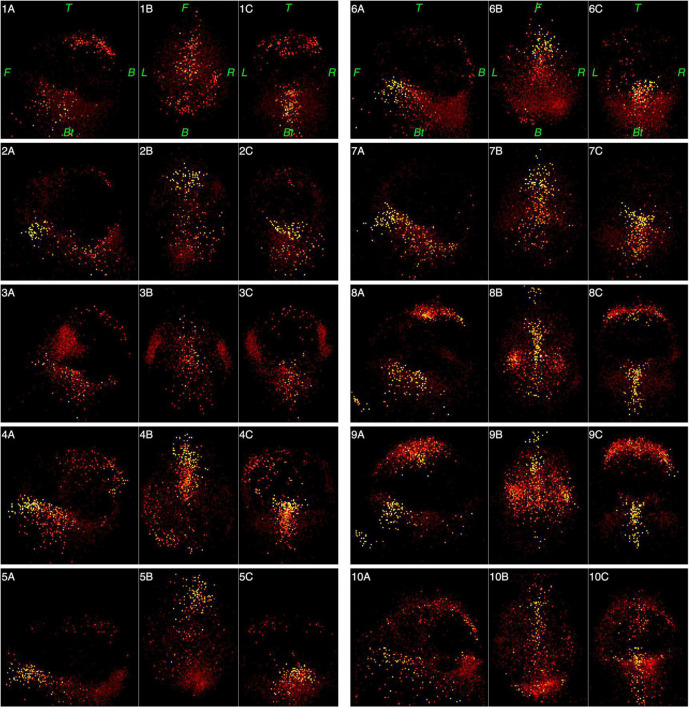
Functional tomograms for all subjects of the «non-brain» areas of the head after the classification of elementary sources, 3D-views: left side view (A); top view (B); frontal view (C). Color map for the power of elementary sources is shown at the bottom of Figure 5. Directions are denoted by: L, left; R, right; F, front; B, back, Bt, bottom; T, top.

Figures 6, 7 represent functional tomograms for all ten subjects. One can see that the same components in various proportions may be distinguished in the non-brain images for each subject: the scalp surface (S), nasal cavity (NC), oral cavity (OC), nasopharynx (Np), larynx (La) and the back of the neck (Nm).

As can be seen from Table 1 and Figure 7, individual variance in the structure and force of the “non-brain” sources is rather high. Still, the “brain” MEG is 10 times higher than “non-brain” MEG even for the wide frequency band 0.3–100 Hz. Cutting out low frequencies drastically improves this ratio to 30 times.

**TABLE 1:** “Brain” to “non-brain” power ratio (BNbR) for all subjects calculated from (17).

Subject number	1	2	3	4	5	6	7	8	9	10	Mean	90% confidence interval
BNbR 0.3–100 Hz	2.9	8.2	29.7	5.3	7.5	5.1	4.4	10.7	7.8	15.5	9.7	5.2–14.2
BNbR 0.3–4.0 Hz	0.3	1.5	21.5	1.2	4.0	3.6	1.2	4.3	5.3	14.6	5.8	1.8–9.7
BNbR 4.0–100 Hz	52.8	50.8	34.1	33.6	56.1	11.5	28.3	24.9	11.2	16.0	31.9	22.3–41.5

**FIGURE 8:**
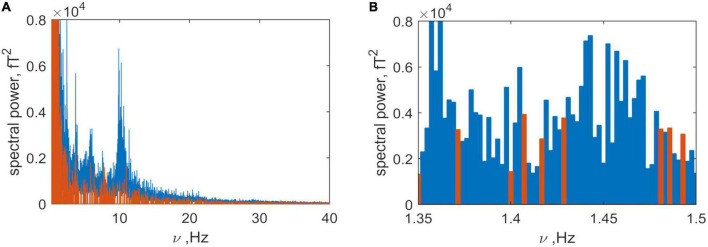
**(A)** Spectra produced by the brain (blue) and non-brain (orange) sources. **(B)** Detail of panel **(A)** in the narrow frequency band 1.15 to 1.3 Hz, illustrating the mix of frequencies.

The next issue addressed concerned the dominant frequencies of the brain and non-brain sources. The spectral power of the brain (blue) and non-brain (orange) sources are plotted as a function of frequency in Figure 8A. Examination of the plot shows that both spectra share very similar frequency bands. This is shown in more detail in Figure 8B where the presence of activity from both sources within a narrow frequency range is illustrated. This means that, without losing information, they cannot be separated using a simple bandpass filter as was used to separate the head signal from that generated by the instrument and far noise (Figure 1). But, without filtering (e.g., of frequencies less than 5 Hz) it is also problematic because non-brain frequencies will be included in the inverse problem solution for the brain.

**FIGURE 9:**
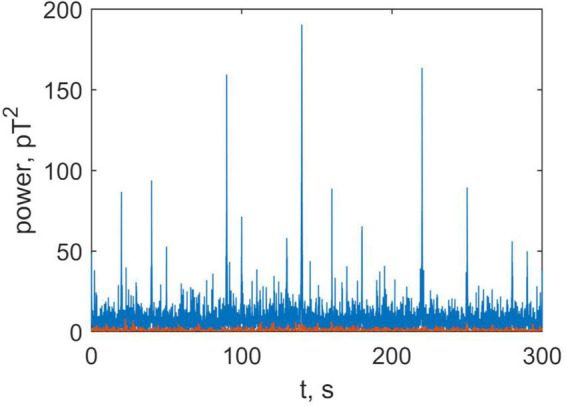
Time series of the summary power in all channels, produced by the brain (blue) and non-brain areas (orange).

To explore this further, we compared the power of the brain and non-brain signals. This is shown in Figure 9 where the instantaneous power produced by brain (blue) and non-brain (orange) sources is plotted as a function of time (see Formulae 13 and 14). This figure illustrates that the brain signal is approximately ten times greater than non-brain signal (physiological noise), and that the former is much more volatile.

**FIGURE 10:**
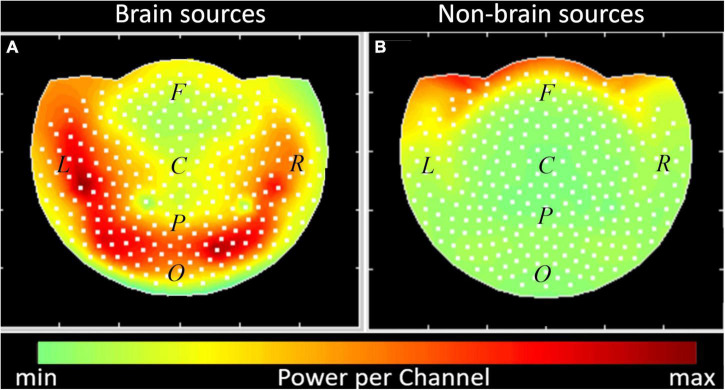
Summary power in channels of registration, produced by the brain **(A)** and non-brain physiological sources **(B)**. Interpolated field maps are presented, the sensors are denoted by white dots. Color map for the summary power per channel is shown along the bottom. Dots show the location of each sensor channel in a stereographic projection of the helmet. Groups of sensors were marked according to the instrument description (F, frontal; C, central; L, left; R, right; P, parietal; O, occipital).

Finally, to characterize the general effect of dividing the sources into brain and non-brain with respect to the recording device we calculated the summary power per channel produced for the duration of experiment (see Formulae 14 and 15). The field maps in Figure 10 give a view of the MEG helmet from above. The white dots represent the location of the sensors. The right side (R), left side (L), and center (C) of the head are indicated. The approximate location of the frontal (F), parietal (P), and occipital (O) regions of the brain are also marked. The cumulative powers as registered by each sensor from all the sources in the brain and non-brain areas are color-coded. While the brain sources are registered by all the sensors (Figure 8A), the non-brain sources are located mainly in the frontal channels (Figure 8B).

## Discussion

As can be concluded from the Table 1 and Figure 7, for low frequencies (0.3–4.0 Hz) the MEG from non-brain sources can be comparable or even greater than such of the brain sources. A detailed study of the spectra and localization of the sources shows that the most powerful non-brain sources are modulated by the breathing and by the heart beat (some experiments demonstrate up to four heartbeat harmonics). It leads to the necessity to carefully consider very low brain frequencies, such as delta rhythm. Some details of the non-brain functional tomograms, such as scalp and neck muscles can be seen at rather high frequencies (up to 100 Hz). Also, the method proposed can be used to clear this signal by removing non-brain frequencies based on their origin.

Such a division into brain and non-brain signal is possible under the assumption that the elementary sources (groups of neurons) are motionless. In some works [see e.g., (Martinet et al., 2017; Zhang et al., 2018; Halgren et al., 2019; Stolk et al., 2019)] the propagation of activity waves during epileptic seizures and alpha rhythm waves were studied using electrocorticography.

The proposed method of functional tomography can be used not only to study stationary sources of spontaneous activity, but also to study the spread of excitation in the event of evoked activity or seizures. To do this, we propose the following modification of the method: the space under study is divided into small volumes, the boundaries of which can be determined both by anatomical sections and in an arbitrary way, each of these volumes becomes a “virtual electrode.” For each of the virtual electrodes, its partial spectrum is found, and a multichannel time series is restored over the entire recording time–a kind of “encephalogram” of the selected area. Then the obtained time series are proposed to be divided into shorter consecutive intervals of 1–10 s, in order to study correlations and cause-and-effect relationships between time series from different virtual electrodes. So, the propagation of activity will be described by the switching between stable elementary sources.

Another possible modification of the method is to calculate functional tomograms over a set of short consecutive time intervals. This will reduce the possible number of sources and coarsen the frequency grid. With this approach, it is possible that in different time windows, sources operating at the same frequency will change their spatial localization. The proposed modifications require a separate thorough study and analysis.

## Conclusion

A novel method of the head partial spectroscopy is introduced. The method further develops the recently proposed functional tomography based on MEG data analysis (Llinás and Ustinin, 2014, 2016; Llinás et al., 2015a,b) and makes it possible to divide experimental magnetoencephalographic data into two synthetic encephalograms: one originating from the brain, another originating from the non-brain physiological sources once the non-physiological sources are eliminated. As was found in Llinás et al. (2020), the whole MEG contains signals from the non-brain physiological sources inside the head, interfering with the analysis of brain activity. In this article, the functional tomograms of the brain and non-brain areas of the head were built, and the corresponding time series were calculated. It opens the possibility to further distinguish the magnetic encephalogram from physiological noises and to make recommendations about optimal positioning of the subject in the instrument or even in devising the helmet’s configuration. Also, a more detailed study of physiological electrical sources in the head is possible, including non-brain areas (e.g., pharynx), and brain areas containing some powerful sources modulated by the heartbeat and possibly close to blood vessels. Such a study can be performed based on the more detailed frequency analysis.

## Data availability statement

The datasets analyzed for this study can be found in the data from “Splitting of the Magnetic Encephalogram into «brain» and «non-brain» Physiological Signals Based on the Joint Analysis of Frequency-Pattern Functional Tomograms and Magnetic Resonance Images” https://doi.org/10.17605/OSF.IO/6Q2JA.

## Ethics statement

The studies involving human participants were reviewed and approved by the NYU Grossman School of Medicine Institutional Review Board. The patients/participants provided their written informed consent to participate in this study.

## Author contributions

RL, MU, and KW designed the research. RL and KW performed the research. RL, MU, SR, KW, and AB analyzed the data. RL, SR, KW, and MU wrote the manuscript. All authors contributed to the article and approved the submitted version.

## Footnotes

1
https://surfer.nmr.mgh.harvard.edu/
